# Supplementary material for: Serum and Muscle 1H NMR-Based Metabolomics Profiles Reveal Metabolic Changes Influenced by a Maternal Leucine-Rich Diet in Tumor-Bearing Adult Offspring Rats
Source: Nutrients. 2020 Jul 16;12(7):2106. doi: 10.3390/nu12072106 (PMC7400806; doi:10.3390/nu12072106)
Supplement: Supplementary file 1 [file nutrients-12-02106-s001.pdf]

nutrients

Support Information

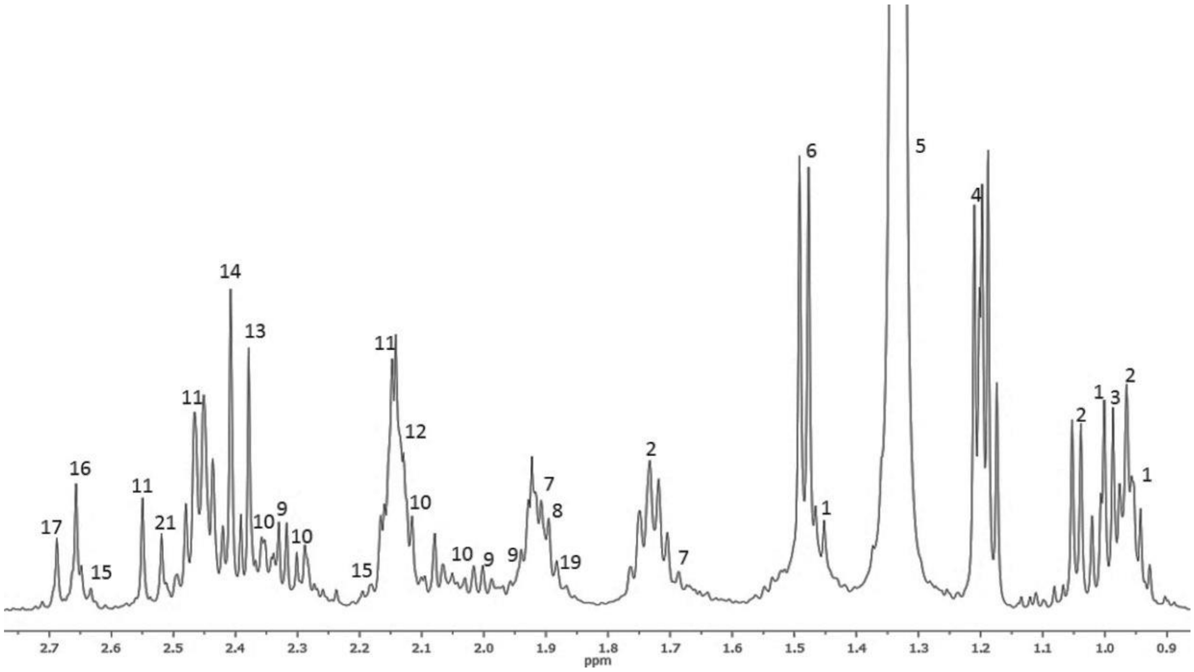

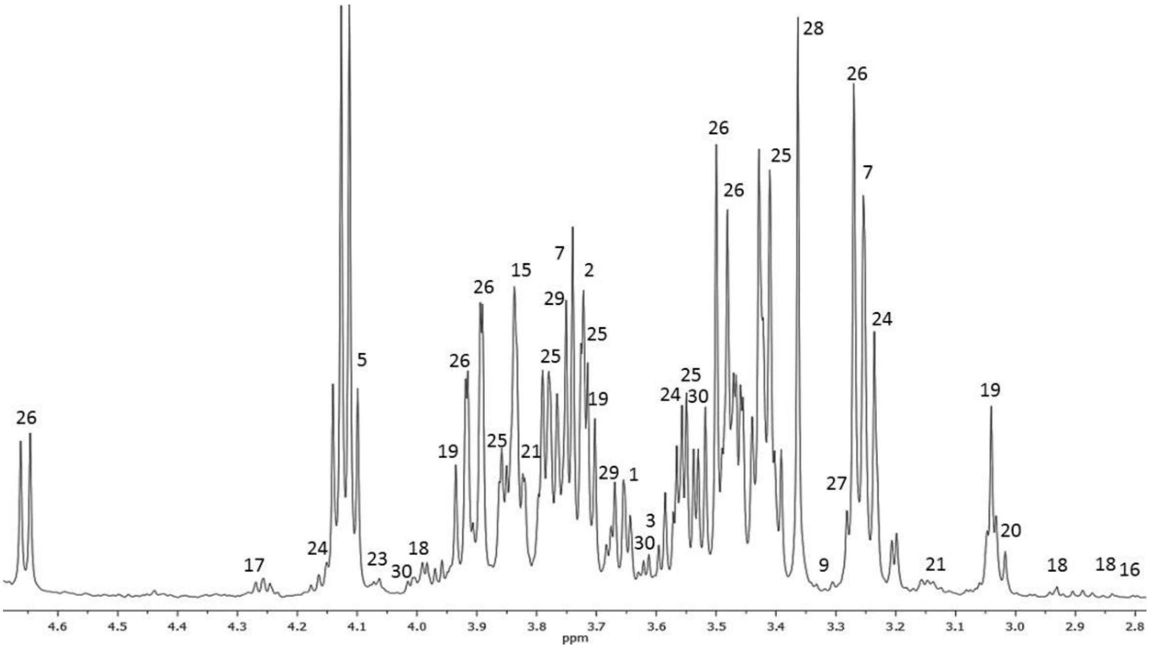

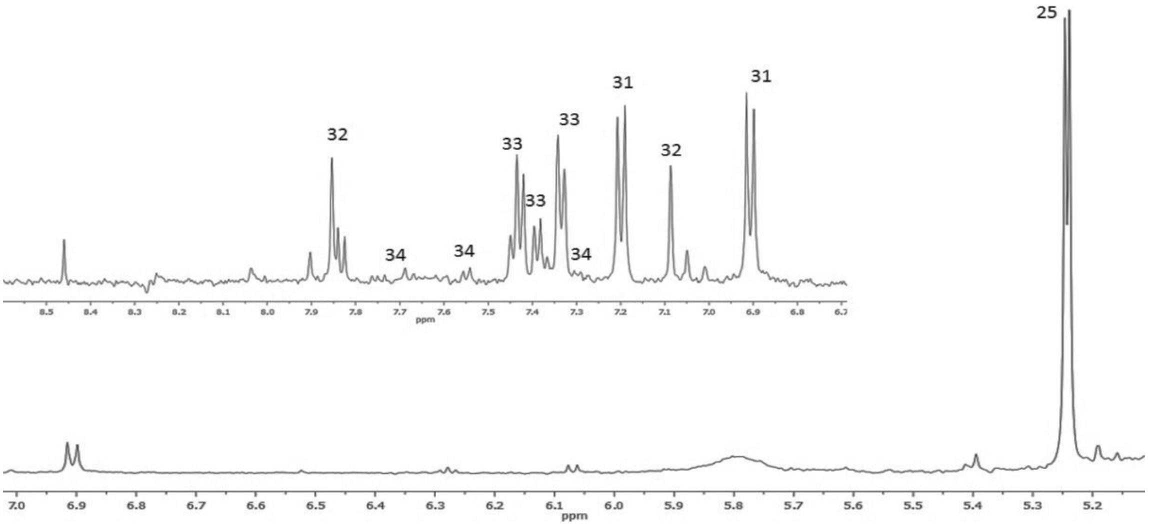

Figure S1. Representative ^{1}H NMR serum spectra of a sample of the control group identifying 34 metabolites, in amplified spectral regions from 5.1–7.1 ppm, 6.7–8,6 ppm, 2.7–4.8 ppm and 0.8–2.9 ppm from the bottom to the top, and numbered peaks of metabolites, which were assigned as shown in Table S1.

Initial spectral analysis of serum sample metabolites

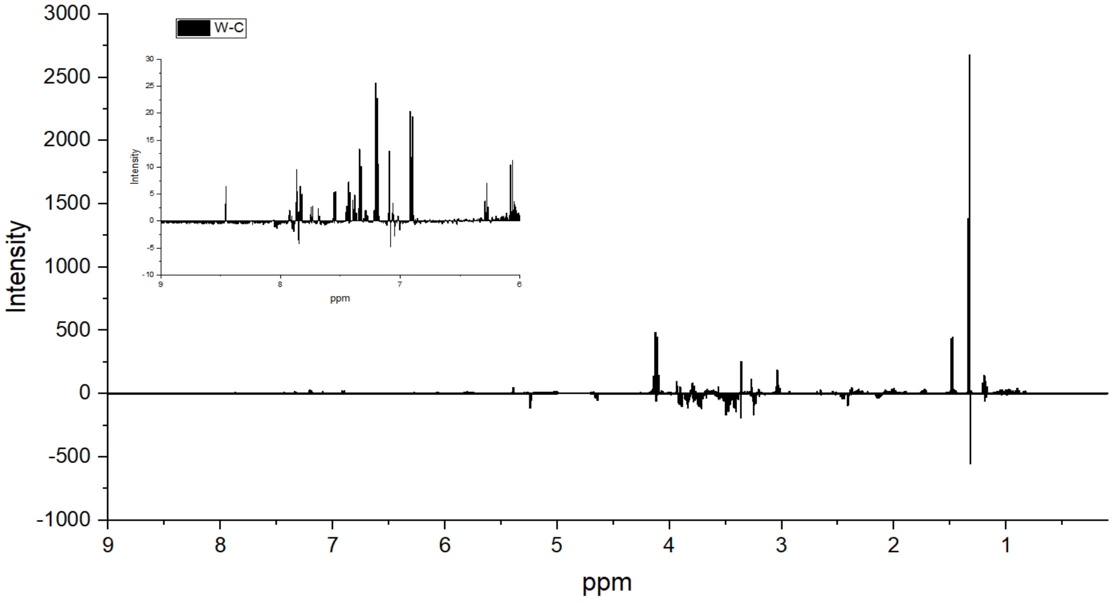

Figure S2. Comparison of ^{1}H NMR data of serum samples between W and C groups to evaluate the tumour effects. The graphic represents the average of the NMR spectra of the W group minus the average of the NMR spectra of the C group. Legend: Tumour-bearing group (W) and Control group (C).

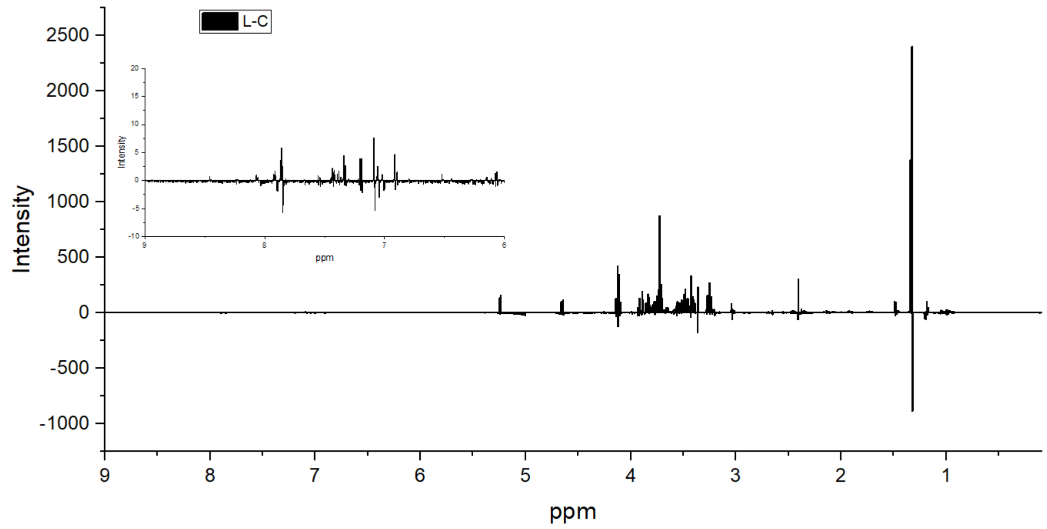

Figure S3. Comparison of ^{1}H NMR data of serum samples between L and C groups to evaluate the maternal diet effect. The graphic represents the average of the NMR spectra of the L group minus the average of the NMR spectra of the C group. Legend: Control group with a maternal leucine-rich diet (L) and Control group (C).

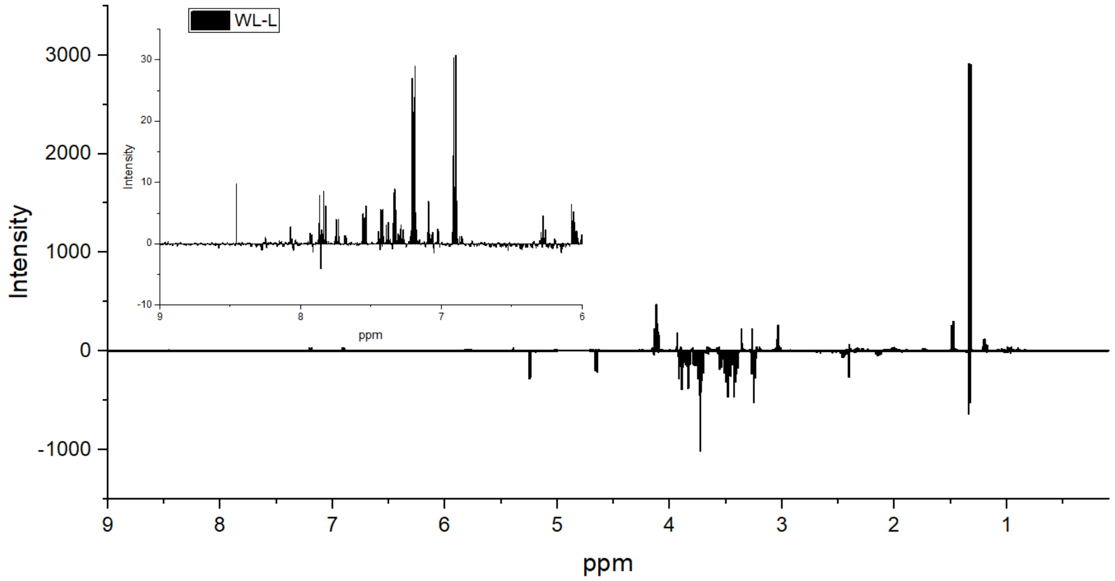

Figure S4. Comparison of ^{1}H NMR data of serum samples between WL and L groups to evaluate the maternal diet effect. The graphic represents the average of the NMR spectra of the WL group minus the average of the NMR spectra of the L group. Legend: Tumour-bearing group with a maternal leucine-rich diet (WL) and Control group with a maternal leucine-rich diet (L).

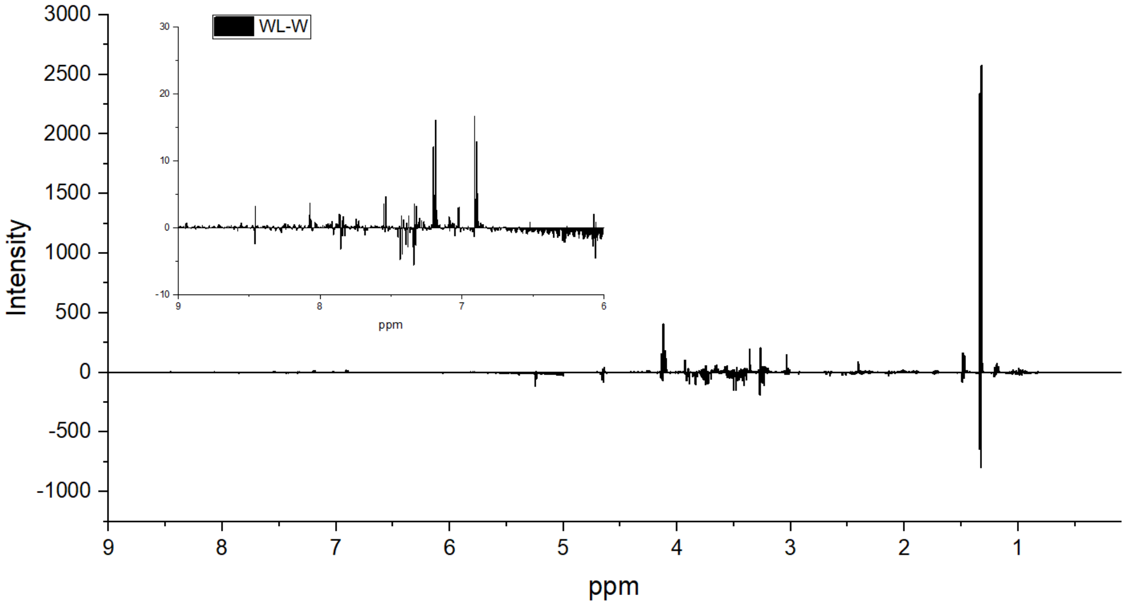

Figure S5. Comparison of ^{1}H NMR data of serum samples between WL and W groups to evaluate the maternal diet effect on cancer development. The graphic represents the average of the NMR spectra of the WL group minus the average of NMR spectra of the W group. Legend: Tumour-bearing group (W) and Tumour-bearing group with a maternal leucine-rich diet (WL).

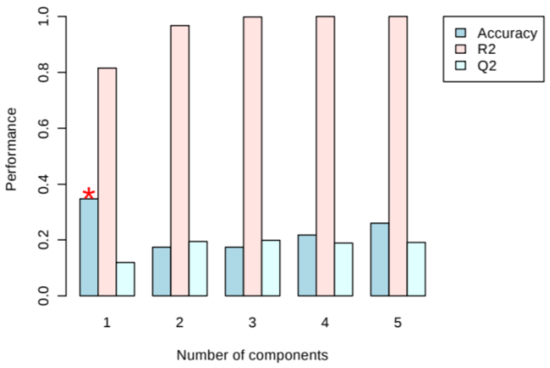

PLS-DA cross validation details:

Measure	1 comps	2 comps	3 comps	4 comps	5 comps
Accuracy	0.34783	0.17391	0.17391	0.21739	0.26087
R2	0.81543	0.96742	0.99801	0.99978	0.99998
Q2	0.11907	0.19407	0.19838	0.1884	0.19066

Figure S6. Accuracy, R2 and Q2 for PLS-DA model for four groups (C; L; W: WL) from serum ^{1}H NMR spectra, generated by MetaboAnalyst, corresponding to the 3D PLS-DA model from Figure 1A. The red asterisk indicates significant differences. For more details, see Material and Method section.

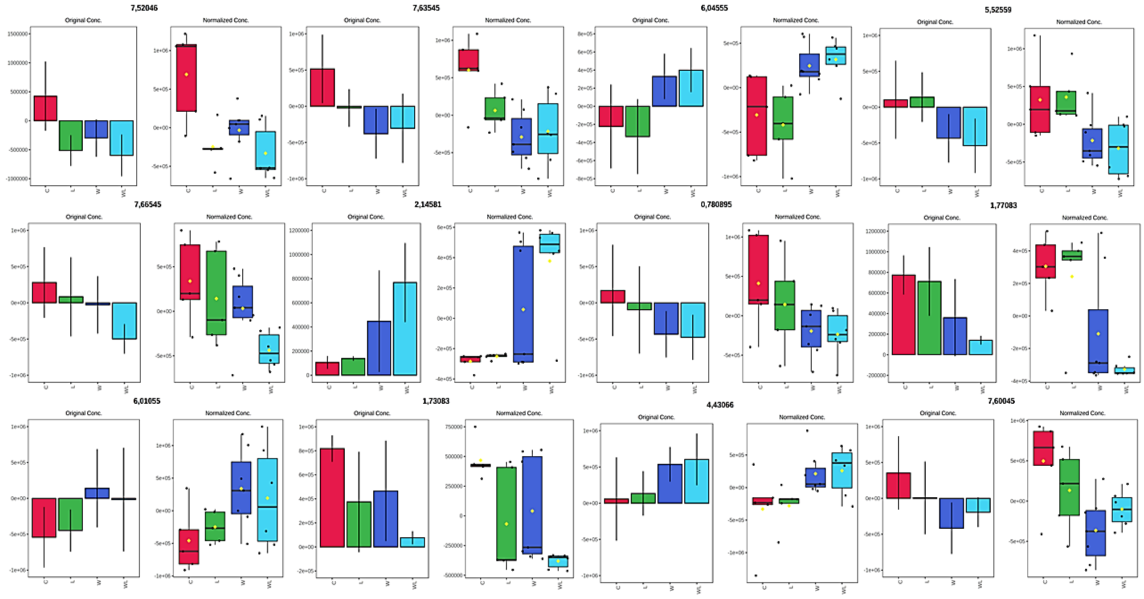

Figure S7. Important variables (VIPs) of peaks chemical assignments extracted from PLS-DA, loadings plots for the experimental groups (C; L; W: WL) from serum ^{1}H NMR spectra. Legend: Control group (C, red), Control group with a maternal leucine-rich diet (L, green), Tumour-bearing group (W, blue) and Tumour-bearing group with a maternal leucine-rich diet (WL, light blue).

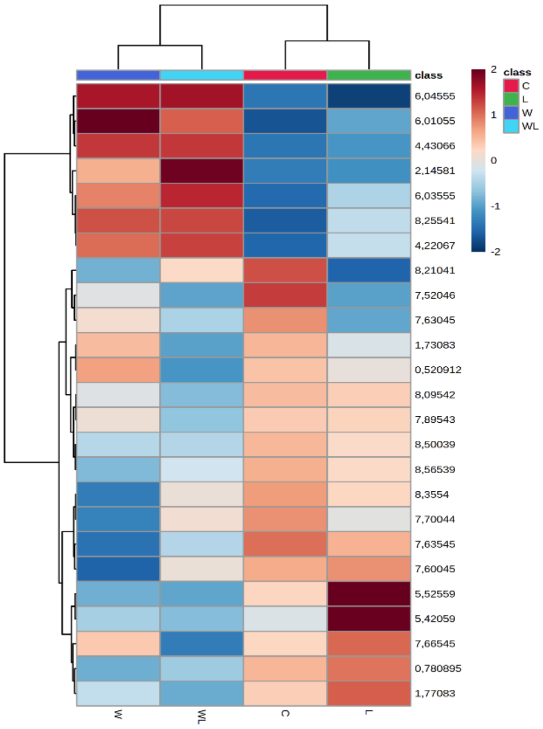

Figure S8. Heatmap plot for PLS-DA model for the experimental groups (C; L; W: WL) from serum 1H NMR spectra. Legend: Control group (C), Control group with a maternal leucine-rich diet (L), Tumour-bearing group (W) and Tumour-bearing group with a maternal leucine-rich diet (WL).

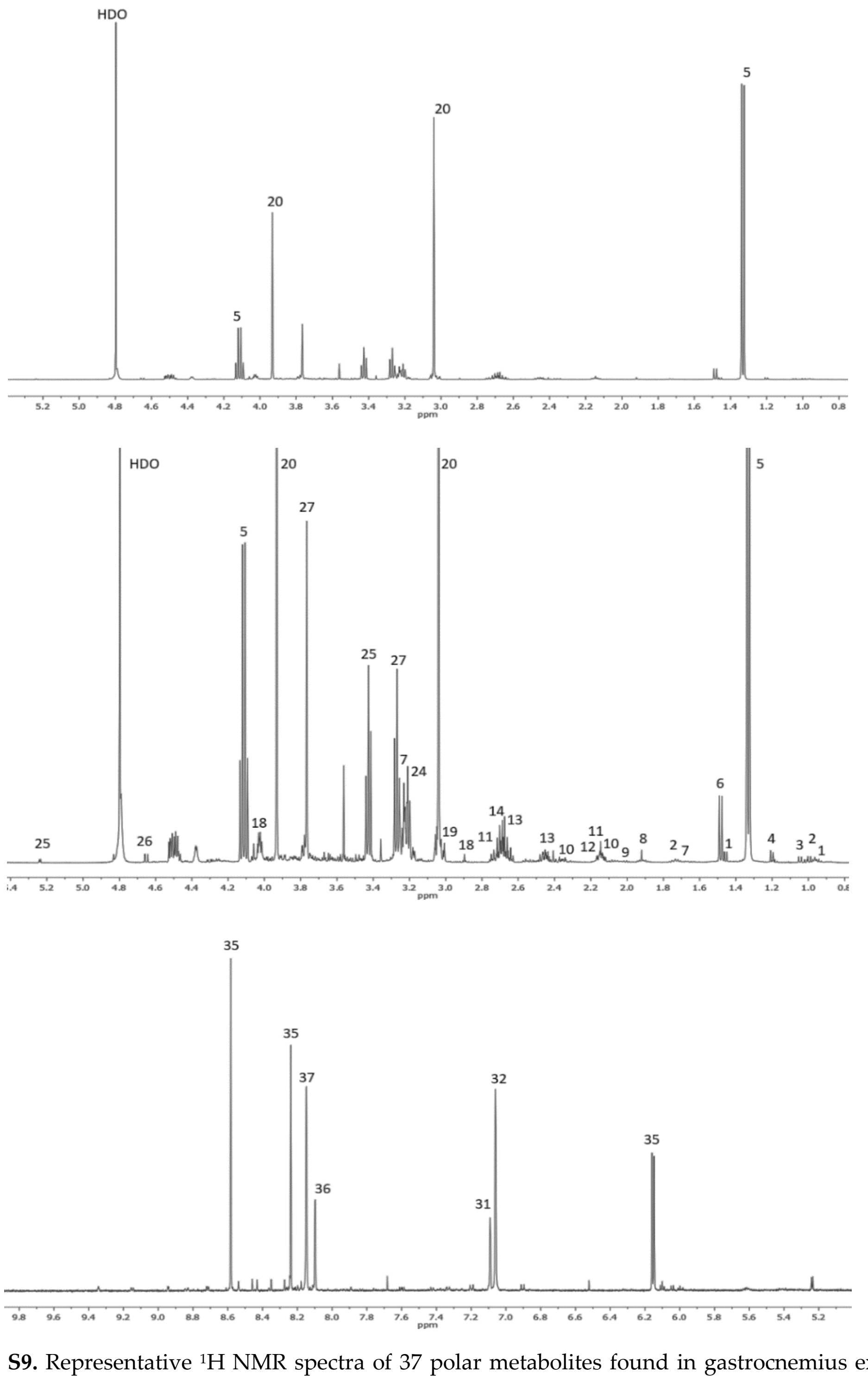

Figure S9. Representative ^{1}H NMR spectra of 37 polar metabolites found in gastrocnemius extract form a sample of the control group, in amplified spectral regions from 5.0–9.9 ppm, 0.8–5.4 ppm and 0.7–5.4 ppm from the bottom to the top, and numbered peaks of metabolites, which were assigned as shown in Table S1.

Initial spectral analysis of polar metabolites from gastrocnemius muscle

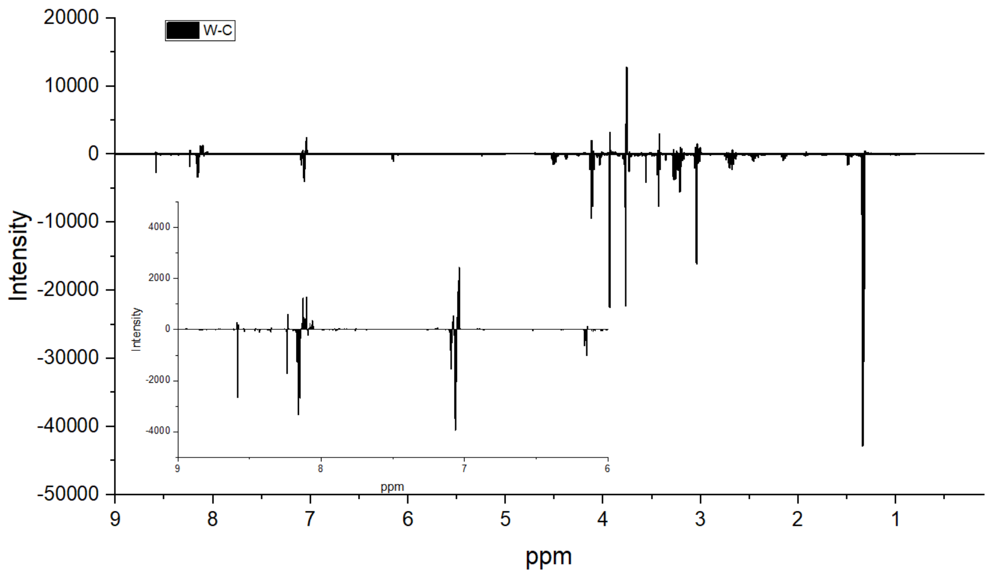

Figure S10. Comparison of ^{1}H NMR data of polar metabolites of gastrocnemius muscle samples between W and C groups to evaluate the tumour effects. The graphic represents the average of the NMR spectra of the W group minus the average of the NMR spectra of the C group. Legend: Tumour-bearing group (W) and Control group (C).

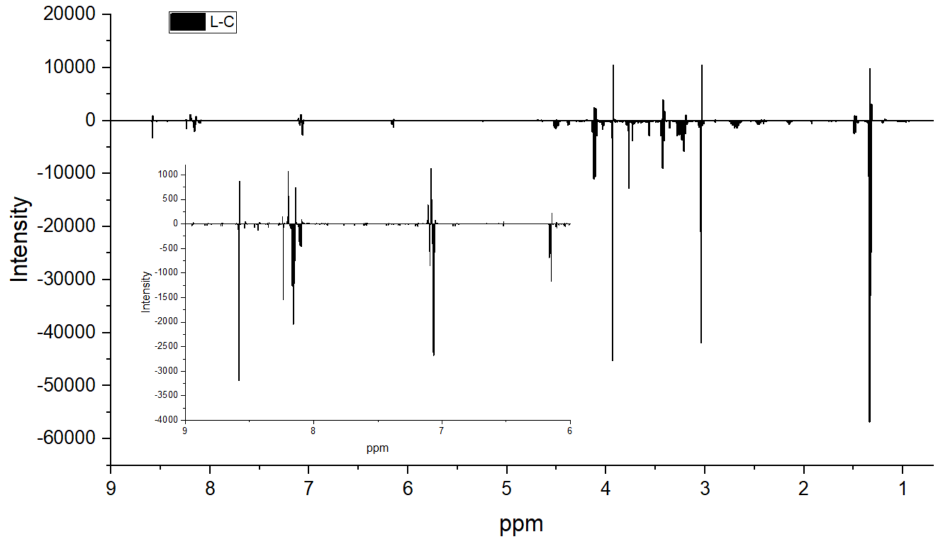

Figure S11. Comparison of ^{1}H NMR data of polar metabolites of gastrocnemius muscle samples between L and C groups to evaluate the maternal diet effect. The graphic represents the average of the NMR spectra of the L group minus average of the NMR spectra of C group. Legend: Control group with a maternal leucine-rich diet (L) and Control group (C).

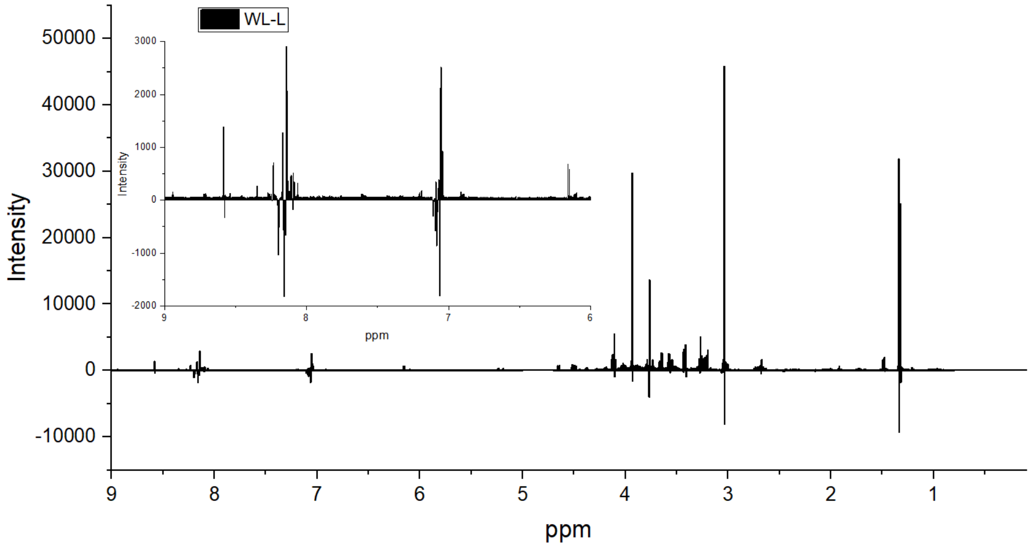

Figure S12. Comparison of ^{1}H NMR data of polar metabolites of gastrocnemius muscle samples between WL and L groups - to evaluate the maternal diet effect. The graphic represents the average of the NMR spectra of the WL group minus the average of NMR spectra of the L group. Legend: Tumour-bearing group with a maternal leucine-rich diet (WL) and Control group with a maternal leucine-rich diet (L).

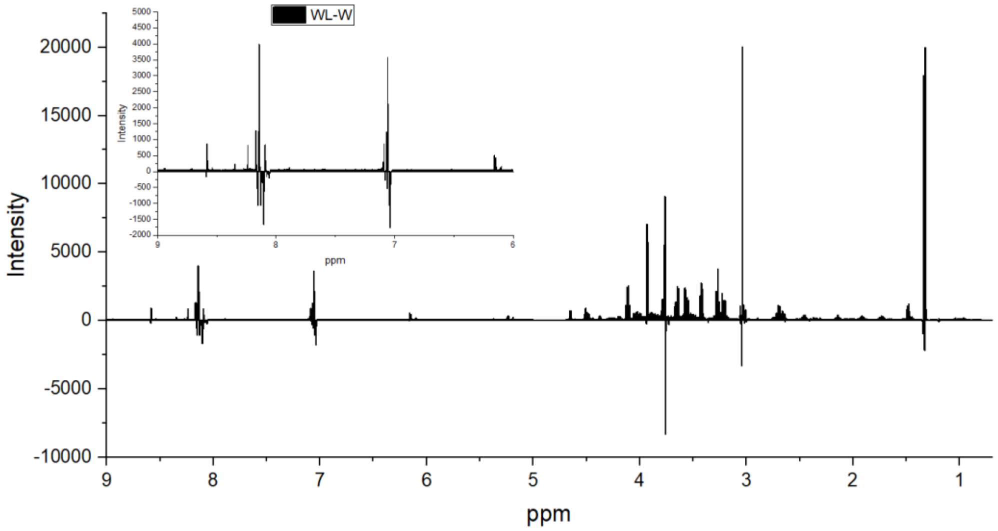

Figure S13. Comparison of ^{1}H NMR data of polar metabolites of gastrocnemius muscle samples between WL and W groups to evaluate the maternal diet effect on cancer development. The graphic represents the average of the NMR spectra of the WL group minus the average of the NMR spectra of the W group. Legend: Tumour-bearing group (W) and Tumour-bearing group with a maternal leucine-rich diet (WL).

Initial spectral analysis of non-polar metabolites from gastrocnemius muscle

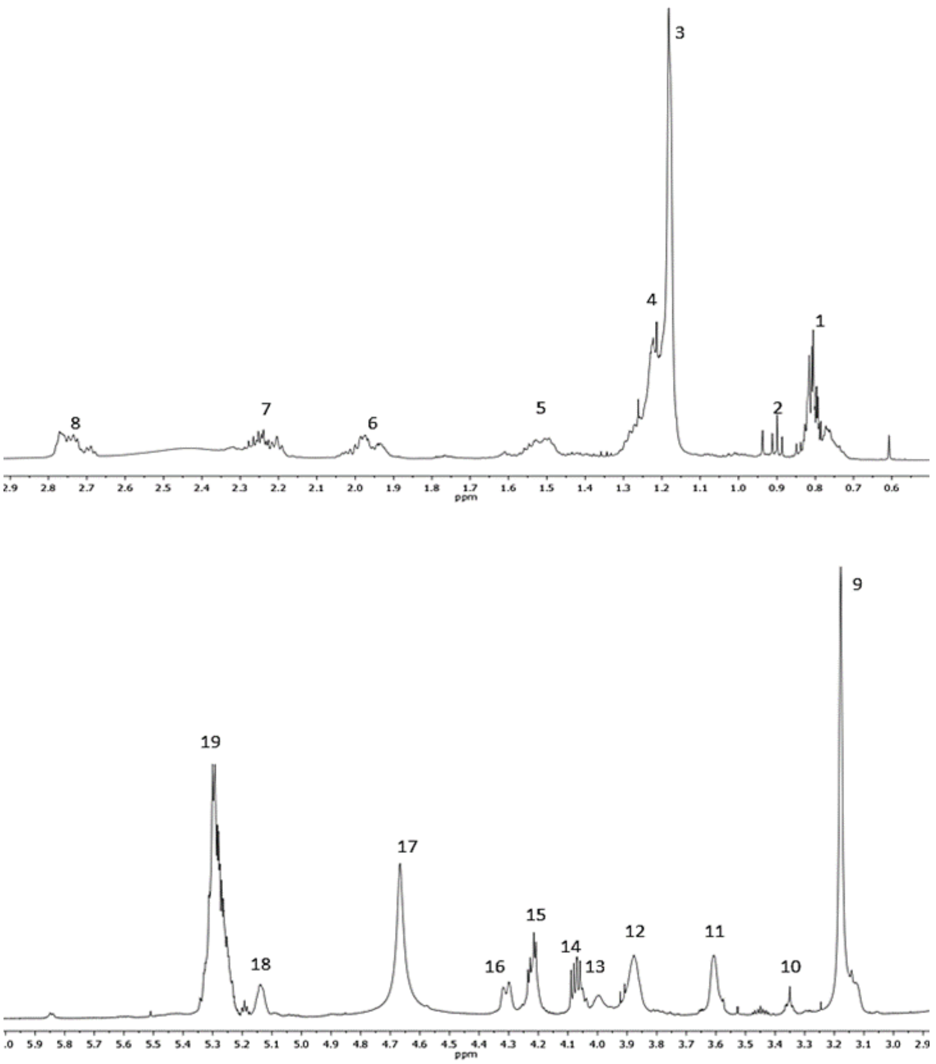

Figure S14. Representative ^{1}H NMR gastrocnemius non-polar muscle spectra of a control group sample with 19 peaks found and assignment from 0.8 to 6.0 ppm.

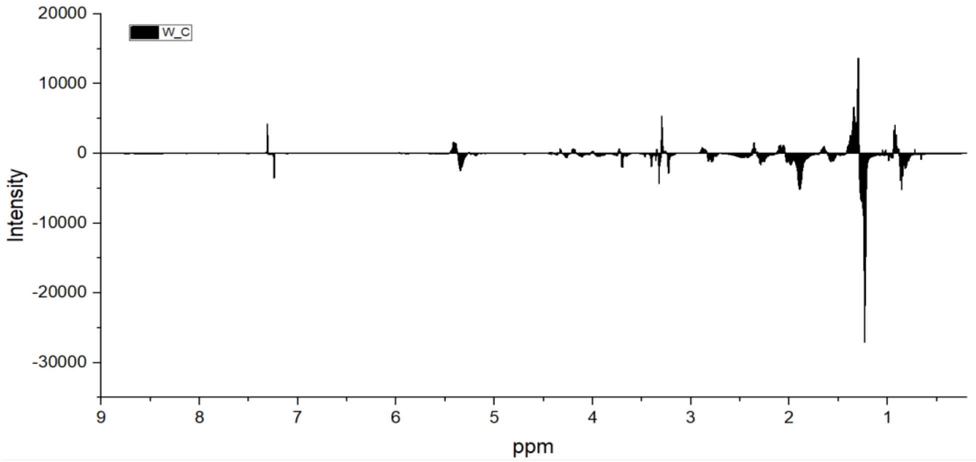

Figure S15. Comparison of ^{1}H NMR data of non-polar metabolites of gastrocnemius muscle samples between W and C groups to evaluate the tumour effects. The graphic represents the average of the NMR spectra of the W group minus the average of the NMR spectra of the C group Legend: Tumour-bearing group (W) and Control group (C).

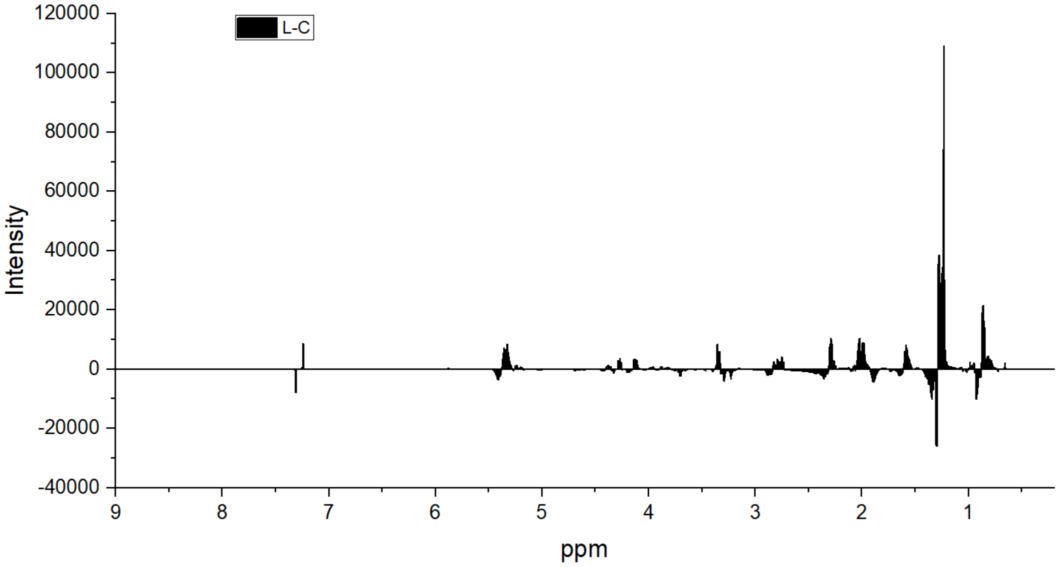

Figure S16. Comparison of ^{1}H NMR data of non-polar metabolites of gastrocnemius muscle samples between L and C groups to evaluate the maternal diet effect. The graphic represents the average of the NMR spectra of the L group minus the average of the NMR spectra of the C group. Legend: Control group with a maternal leucine-rich diet (L) and Control group (C).

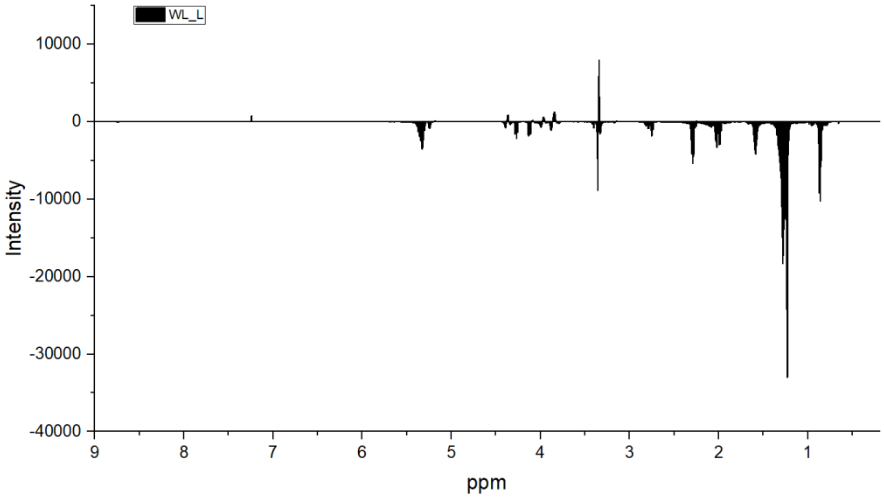

Figure S17. Comparison of ^{1}H NMR data of non-polar metabolites of gastrocnemius muscle samples between WL and L groups to evaluate the maternal diet effect. The graphic represents the average of the NMR spectra of the WL group minus the average of the NMR spectra of the L group. Legend: Tumour-bearing group with a maternal leucine-rich diet (WL) and Control group with a maternal leucine-rich diet (L).

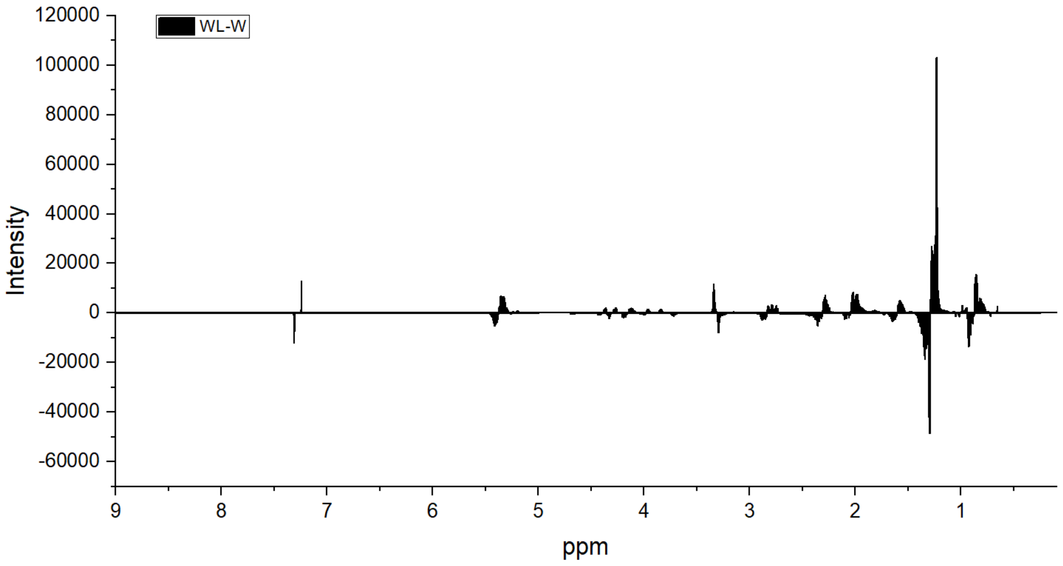

Figure S18. Comparison of ^{1}H NMR data of non-polar metabolites of gastrocnemius muscle samples between WL and W groups to evaluate the maternal diet effect on cancer development. The graphic represents the average of the NMR spectra of the WL group minus average of NMR spectra of the W group. Legend: Tumour-bearing group (W) and Tumour-bearing group with a maternal leucine-rich diet (WL).

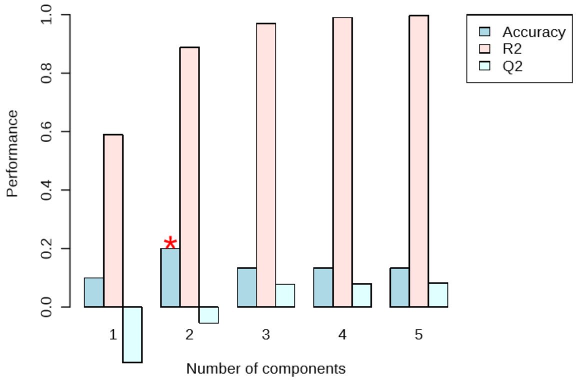

PLS-DA cross validation details:

Measure	1 comps	2 comps	3 comps	4 comps	5 comps
Accuracy	0.16667	0.13333	0.16667	0.1	0.13333
R2	0.56645	0.89199	0.96027	0.98484	0.99602
Q2	-0.1049	0.08295	0.10064	0.13423	0.13888

Figure S19. Accuracy, R 2and Q 2for PLS-DA model for the experimental groups (C; L; W: WL) from polar gastrocnemius extract ^{1}H NMR spectra. The red asterisk indicates significant differences. For more details, see Material and Method section.

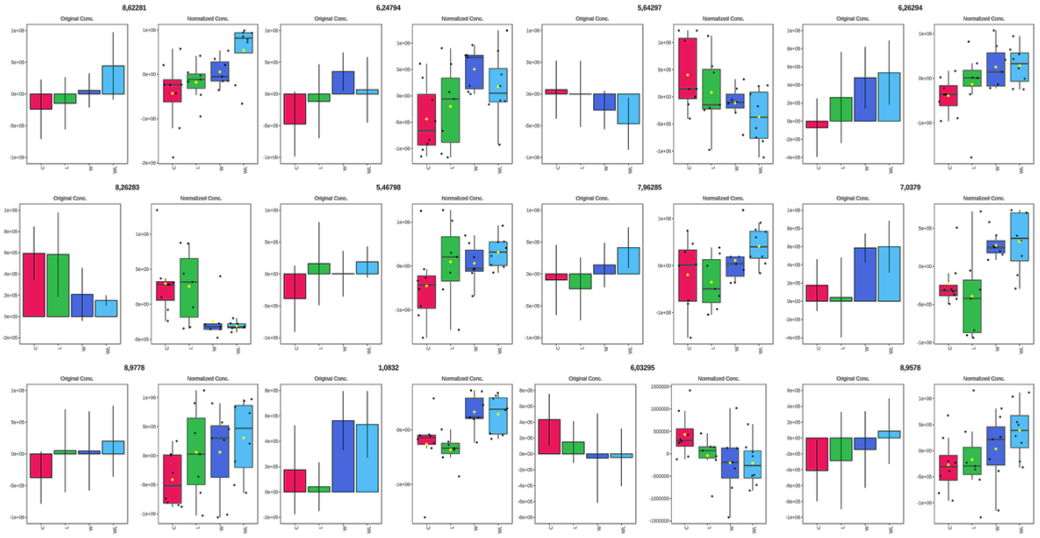

Figure S20. Important variables (VIPs), peaks chemical assignments extracted from PLS-DA loadings plots for the experimental groups (C; L; W: WL) from polar gastrocnemius extract of ^{1}H NMR spectra. Legend: Control group (C, red), Control group with a maternal leucine-rich diet (L, green), Tumour-bearing group (W, blue) and Tumour-bearing group with a maternal leucine-rich diet (WL, light blue).

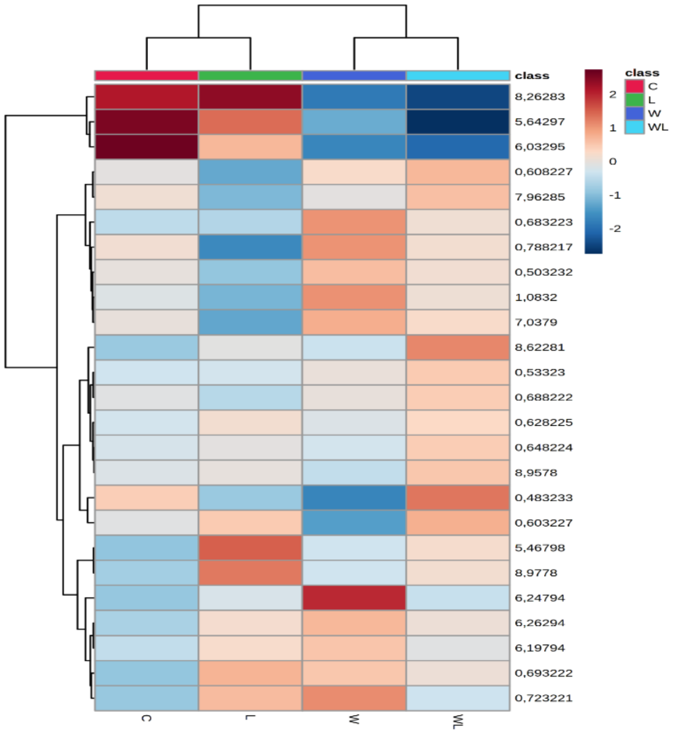

Figure S21. Heatmap plot for PLS-DA VIPs for the experimental groups (C; L; W: WL) generated for polar gastrocnemius extract ^{1}H NMR spectra. Legend: Control group (C), Control group with a maternal leucine-rich diet (L), Tumour-bearing group (W) and Tumour-bearing group with a maternal leucine-rich diet (WL).

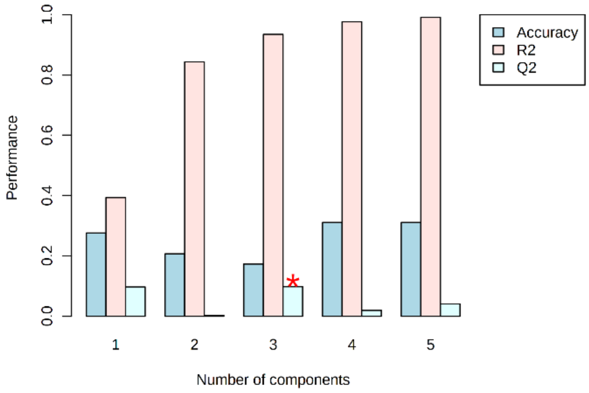

PLS-DA cross validation details:

Measure	1 comps	2 comps	3 comps	4 comps	5 comps
Accuracy	0.27586	0.2069	0.17241	0.31034	0.31034
R2	0.39291	0.84377	0.93493	0.97619	0.99102
Q2	0.096321	0.0026702	0.097411	0.019073	0.040213

Figure S22. Accuracy, R2 and Q2 for PLS-DA model for the experimental groups (C; L; W: WL) from nonpolar gastrocnemius extract ^{1}H NMR spectra. The red asterisk indicates significant differences. For more details, see Material and Method section.

Table S1. Serum and muscle polar metabolites, and their chemical shifts, spectral peak multiplicities and coupling constants found in samples analysed for all experimental groups, shown as 1–37 peaks in Figures S1 and S9.

Metabolite	No.	Chemical Shift (ppm), Spectral Peaks Multiplicities and Coupling Constant
Isoleucine	1	0.926 t (*J*=8 Hz); 0.997 d (*J*=9 Hz); 1.248 m; 1.457 m; 1.968 m; 3.661 d (*J*=4 Hz);
Leucine	2	0.948 t (*J*=8 Hz); 1.71 m; 3.72 m;
Valine	3	0.978 d (*J*=8 Hz); 1.029 d (*J*=7 Hz); 2.261 m (*J*=14, 7, 4.41 Hz); 3.601 d (*J*=4 Hz);
3-Hydroxy butyrate	4	1.204 d (*J*=6.26 Hz); 2.314 m; 2.414 m; 4.160 m;
Lactate	5	1.32 d (*J*=6.96 Hz); 4.12 q (*J*=6.93 Hz);
Alanine	6	1.47 d (*J*=7.28 Hz); 3.77 q (*J*=7.28 Hz);
Arginine	7	1.68 m (*J*=7 Hz); 1.90 m (*J*=7.28 Hz); 3.23 t (*J*=6.93 Hz); 3.76 t (*J*=6.11 Hz);
Acetate	8	1.91 s;
Proline	9	1.99 m (*J*=3 Hz); 2.06 m (*J*=4 Hz); 2.34 m (*J*=4 Hz); 3.33 dt (*J*=14.02, 7.11 Hz); 3.41 dt (J= 11.65, 7.02 Hz); 4.12 dd (J=8.63, 6.42 Hz);
Glutamate	10	2.04 m; 2.119 m; 2.341 m; 3.748 dd (*J*=7.186, 4.724 Hz);
Glutathione	11	2.15 m; 2.54 m; 2.97 dd (*J*=14.23, 9.47 Hz); 3.78 m; 4.20 q (*J*=7.14 Hz);
Glutamine	12	2.125 m; 2.446 m; 3.766 t (*J*=6.18 Hz);
Pyruvate	13	2.46 s;
Succinate	14	2.39 s;
Methionine	15	2.157 m; 2.631 t (*J*=7.587 Hz); 3.851 dd (*J*=7.1, 5.382 Hz);
Aspartate	16	2.66 dd (*J*=17.45, 8.85 Hz); 2.80 dd (*J*=17.45, 3.72 Hz); 3.89 dd (*J*=8.82, 3.75 Hz);
Malate	17	2.35 dd (*J*=15.37, 10.24 Hz); 2.66 dd (*J*=15.37, 2.99 Hz); 4.29 dd (*J*=10.23, 2.98 Hz);
Asparagine	18	2.84 m; 2.94 m; 4.00 dd (*J*=7.69, 4.26 Hz);
Lysine	19	1.46 m; 1.71 m; 1.89 m; 3.02 t; 3.74 t (*J*=6.09 Hz);
Creatine	20	3.02 s; 3.92 s;
Ethanolamine	21	3.13 t; 3.83 t;
β-Alanine	22	2.53 t; 3.17 t;
Choline	23	3.189 s; 3.507 dd (*J*=5.816, 4.162 Hz); 4.056 ddd;
O-Phosphocholine	24	3.21 s; 3.58 m; 4.16 m;
α-Glucose	25	3.23 dd (*J*=9.41, 7.98 Hz); 3.40 m; 3.46 m; 3.52 dd (*J*=9.82, 3.77); 3.73 m; 3.82 m; 3.88 dd (*J*=12.30, 2.23 Hz); 4.63 d (*J*=7.98); 5.22 d (*J*=3.80);
β-Glucose	26	3.25 m; 3.49 m; 3.50 m; 3.88 m; 3.91 m; 4.66 d;
Betaine	27	3.25 s; 3.89 s;
Methanol	28	3.33 s;
Mannitol	29	3.649 dd (*J*=11.76, 6.26 Hz); 3.729 m; 3.771 d; 3.840 dd (*J*=11.87, 2.86 Hz);
Myo-Inositol	30	3.268 t (*J*=11.76 Hz); 3.524 dd (*J*=9.978, 2.867); 3.613 t (*J*=9.702 Hz); 4.053 t (*J*=2.839 Hz);
Tyrosine	31	3.03 dd (*J*=14.55, 8.01 Hz); 3.34 dd (*J*=14.53, 4.68Hz); 4.04 dd (*J*=8.03, 4.68 Hz); 6.94 m; 7.20 dd (*J*=7.95, 1.51 Hz); 7.24 td (*J*=7.76, 1.71 Hz);

Histidine	32	3.16 dd (*J*=15.55, 7.75 Hz); 3.23 dd (*J*=16.10, 4.93 Hz); 3.98 dd (*J*=7.73, 4.98 Hz); 7.09 d (*J*=0.58 Hz); 7.90 d (*J*=1.13 Hz);
Phenylalanine	33	3.19 m; 3.98 dd (*J*=7.88, 5.31 Hz); 7.32 d (*J*=6.96 Hz); 7.36 m; 7.42 m;
Tryptophan	34	3.292 dd (*J*=15.353, 8.076 Hz); 3.472 dd (*J*=15.38, 4.796 Hz); 4.046 dd (*J*=8.104, 4.851 Hz); 7.194 m; 7.274; 7.310 s; 7.531 d (*J*=8.159 Hz); 7.723 d (*J*=7.993 Hz);
AMP	35	4.01 m; 4,36 m; 4.50 q; 4.79 t; 6.12 d; 8.25 s; 8.58 s;
ATP	36	4.23 m; 4.27 m; 4.56 t; 4.73t; 6.12 d; 8.25 s; 8.49 s;
IMP	37	4.03 m; 4.36 m; 4.49 t;

Table S2. Total lipids presented in gastrocnemius muscle non-polar extract and their chemical shifts, corresponding to the Figure S14.

**Assignments**	**No.**	**Chemical shift (ppm)**
-$\mathbf{CH_3}$ terminal protons of fatty acyl chains and cholesterol	1	0.55–0.85
-$\mathbf{CH_3}$ protons of ω-3 unsaturated fatty acids	2	0.90–1.05
cholesterol	3	1.15–1.20
-($\mathbf{CH_2}$)n methylene protons of aliphatic chains	4	1.20–1.30
-OCO-CH_2-$\mathbf{CH_2}$ β methylene protons associated to carbonyl groups	5	1.47–1.60
$\mathbf{CH_2}$-CH=CH- α methylene protons associated to double bonds	6	1.90–2.05
-OCO-$\mathbf{CH_2}$ α methylene protons associated to carbonyl groups	7	2.19–2.30
=CH-$\mathbf{CH_2}$-CH= divinyl methylene protons of ω-3 and ω-6 unsaturated fatty acids	8	2.75–2.85
sphingomyelin and choline	9	3.19
-N^+ ($\mathbf{CH_3}$) in phosphatidylcholine, choline and sphingomyelin	10	3.35
glycerophospholipids	11	3.55–3.65
-$\mathbf{CH_2}$-N^+(CH_3) of phosphatidylcholine	12	3.85–3.90
-$\mathbf{CH_2}$-OP in glycerophospholipids	13	3.98
-$\mathbf{CH_2}$ in glycerol chain of phospholipids	14	4.08–4.11
-$\mathbf{CH_2}$ in glycerol chain from triacyclglycerols	15	4.18–4.25
-$\mathbf{CH_2}$ in glycerol chain from triacyclglycerols	16	4.30
-**CH** in esterified group from cholesterol	17	4.65
-**CH**-O from lipid esters	18	5.10–5.15
-**CH**=**CH**- protons in double bonds in unsaturated fatty acids and -**CH** from cholesterol	19	5.20–5.35
